# Multi-scale agent-based brain cancer modeling and prediction of TKI treatment response: Incorporating EGFR signaling pathway and angiogenesis

**DOI:** 10.1186/1471-2105-13-218

**Published:** 2012-08-30

**Authors:** Xiaoqiang Sun, Le Zhang, Hua Tan, Jiguang Bao, Costas Strouthos, Xiaobo Zhou

**Affiliations:** 1 Department of Radiology, The Methodist Hospital Research Institute, Weil Cornell Medical College, Houston, TX, 77030, USA; 2 School of Mathematical Sciences, Beijing Normal University, Beijing, 100875, P R China; 3 College of Computer and Information Science, Southwest University, Chongqing, 400715, P R China; 4 Department of Mathematical Sciences, Michigan Technological University, Houghton, MI, 49931, USA; 5 College of Global Change and Earth System Science, Beijing normal University, Beijing, 100875, P R China; 6 Computation-based Science and Technology Research Center, The Cyprus Institute, Nicosia, 1645, Cyprus

**Keywords:** Multi-scale, Agent-based modeling, EGFR signaling pathway, Angiogenesis, TKI treatment

## Abstract

**Background:**

The epidermal growth factor receptor (EGFR) signaling pathway and angiogenesis in brain cancer act as an engine for tumor initiation, expansion and response to therapy. Since the existing literature does not have any models that investigate the impact of both angiogenesis and molecular signaling pathways on treatment, we propose a novel multi-scale, agent-based computational model that includes both angiogenesis and EGFR modules to study the response of brain cancer under tyrosine kinase inhibitors (TKIs) treatment.

**Results:**

The novel angiogenesis module integrated into the agent-based tumor model is based on a set of reaction–diffusion equations that describe the spatio-temporal evolution of the distributions of micro-environmental factors such as glucose, oxygen, TGFα, VEGF and fibronectin. These molecular species regulate tumor growth during angiogenesis. Each tumor cell is equipped with an EGFR signaling pathway linked to a cell-cycle pathway to determine its phenotype. EGFR TKIs are delivered through the blood vessels of tumor microvasculature and the response to treatment is studied.

**Conclusions:**

Our simulations demonstrated that entire tumor growth profile is a collective behaviour of cells regulated by the EGFR signaling pathway and the cell cycle. We also found that angiogenesis has a dual effect under TKI treatment: on one hand, through neo-vasculature TKIs are delivered to decrease tumor invasion; on the other hand, the neo-vasculature can transport glucose and oxygen to tumor cells to maintain their metabolism, which results in an increase of cell survival rate in the late simulation stages.

## Background

Brain cancer is a very complex and deadly disease. Traditional diagnoses and treatments of this disease are from *in vitro* experimental observations. Although biologists have developed many experimental data at the molecular, cellular, micro-environmental and tissue scales, only very few scientists have integrated these data into multi-scale models to study tumor response to treatment.

*Cellular automata* (*CA*) methods have been widely applied to model brain tumor growth [1,2]. Although *CA* models are good at describing cell-cell and cell-microenvironment interactions, this type of discrete modelling approach falls short on investigating most fluid dynamic aspects of the tumor microenvironment. Alternatively, *Continuum models* employ systems of partial differential equations to simulate the solid tumor invasion by updating boundaries of different sub-domains of tumor based on the level-set method [3,4]. It is, however, hard with this approach to describe cell-cell interactions, such as the competition among cells for nutrients. In general neither continuum nor discrete models can accurately simulate cancer spatio-temporal evolution with respect to the complexity of cancer. A hybrid discrete-continuum (*HDC*) model that couples a cellular automaton module with a continuum module was proposed by Anderson and co-workers [5,6]. Although these models have not considered the effects of any gene-protein signaling pathways such as the EGFR pathway, computational cancer biologists have already studied them extensively. Recent studies showed that EGFR pathway plays an important role in the evolution of brain cancer [7-9]. Therefore, the existing multi-scale agent-based tumor models incorporated an EGFR signaling pathway [10,11] at the molecular scale to enable individual cells to choose their phenotypic trait between proliferation and migration based on the pathway's state [10,12,13]. As indicated by Hanahan and Weinberg[14], angiogenesis is a significant transforming phase in tumor growth. During the angiogenesis phase, tumor cells secret vascular endothelial growth factors (VEGF) [15,16] into the microenvironment to induce and sustain new capillary sprouts migrating from pre-existing vasculature towards the tumor. This in turn helps to maintain tumor cells’ metabolism by supplying them with glucose and oxygen and subsequently leads to metastasis. However, our previous agent-based models [10-13,17] did not explicitly take tumor-induced angiogenesis into consideration.

In this paper, we presented a novel multi-scale agent-based model to describe tumor growth with angiogenesis and study the response of brain cancer to EGFR tyrosine kinase inhibitors (TKIs) [18]. Several rates of changes of molecular species, such as PLCγ, CDh1 and cycCDK will determine a tumor cell’s phenotypic switch.

In order to integrate an angiogenesis module into the existing agent-based tumor growth models [10,11,13,17], we developed a set of rules that underline the migration of endothelial cells and the branching of vessel sprouts. Compared to previous developed HDC rules [19], these rules which directly defined the probabilities of migration of endothelia cells are more suitable for implementation into an agent-based model. The tumor growth and angiogenesis are coupled through VEGF secreted by the tumor cells and through the glucose and oxygen permeated from the neo-vasculature. As the neo-vasculature develops, glucose and oxygen penetrate from the blood vessels and diffuse throughout the tumor microenvironment to promote further tumor growth, which in turn influences the concentration of VEGF.

The simulation results demonstrate that we can investigate the response of brain cancer to tyrosine kinase inhibitors (TKIs) and also can use the model to reveal the dual role of angiogenesis.

## Implementation

**Figure 1:**
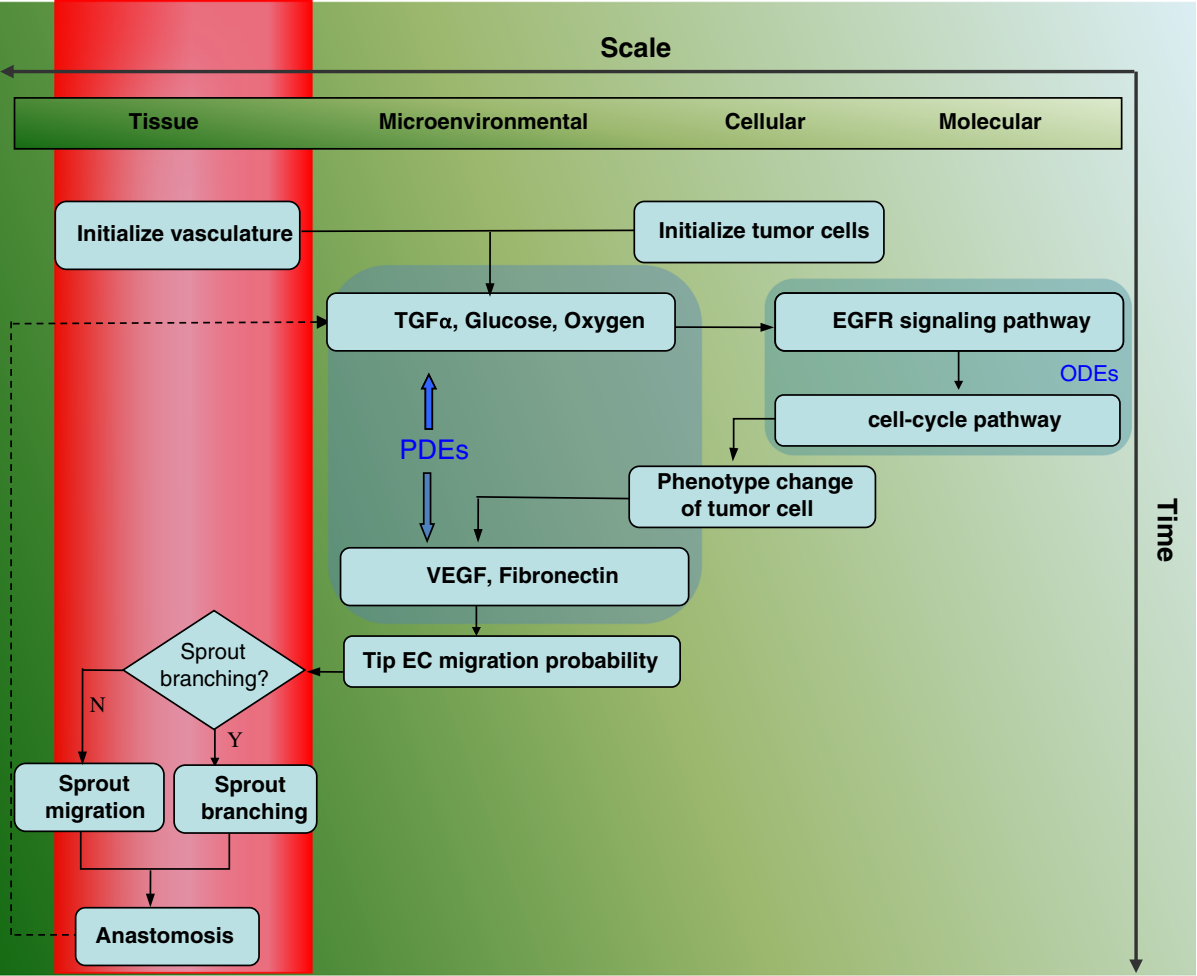
**Flow chart of multi-scale agent-based cancer modeling.** The model encompasses four biological scales: the molecular scale, the cellular scale, the micro-environmental scale and the tissue scale. The molecular scale consists of the EGFR signaling and cell cycle pathways (Figure 2). The cellular scale simulates the phenotypic switch of the tumor cells as determined by the molecular scale, the cell-cell and the cell-microenvironment interactions (Figure 3). The micro-environmental scale provides a connection between the micro-vasculature (angiogenesis) and the tumor cells. Tumor cells consume glucose and oxygen nutrients. The tumor growth factor alpha (TGFα) secreted by tumor cells triggers the EGFR signaling pathway, which in turn determines cell’s phenotype switch. Oxygen plays an important role in the cell cycle during the proliferation phase. The VEGF secreted by the tumor cells induces angiogenesis. The tissue scale focuses on angiogenesis (Figure 4). Blood vessel sprouts migrate and branch via tip endothelial cells' migration in response to VEGF chemotaxis and fibronectin haptotaxis.

**Figure 2:**
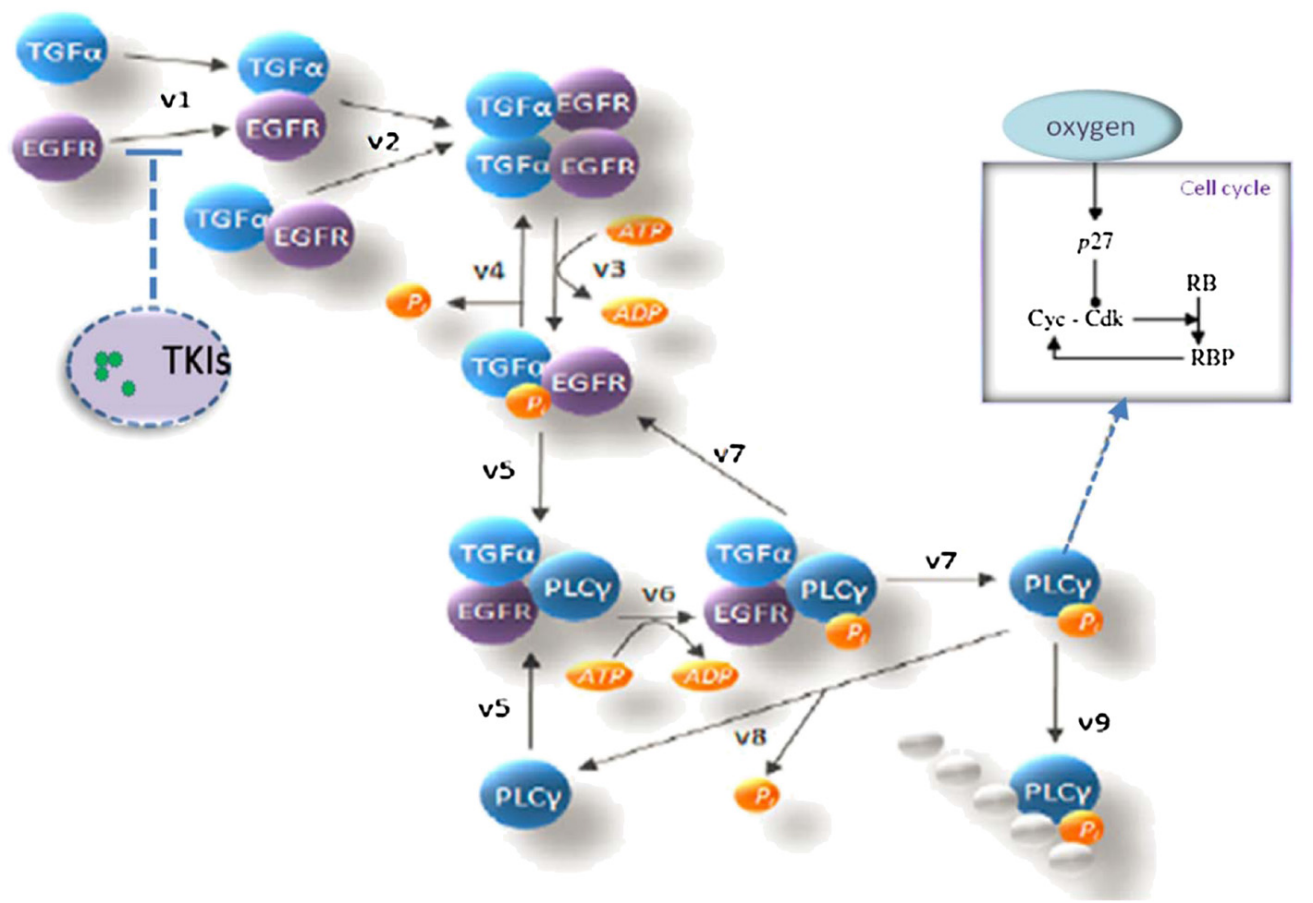
**EGFR signaling pathway connected to cell cycle pathway with TKIs to block the EGFR signaling pathway.** EGFR signaling pathway is connected to the cell cycle pathway, and TKIs block the EGFR signaling pathway.

**Figure 3:**
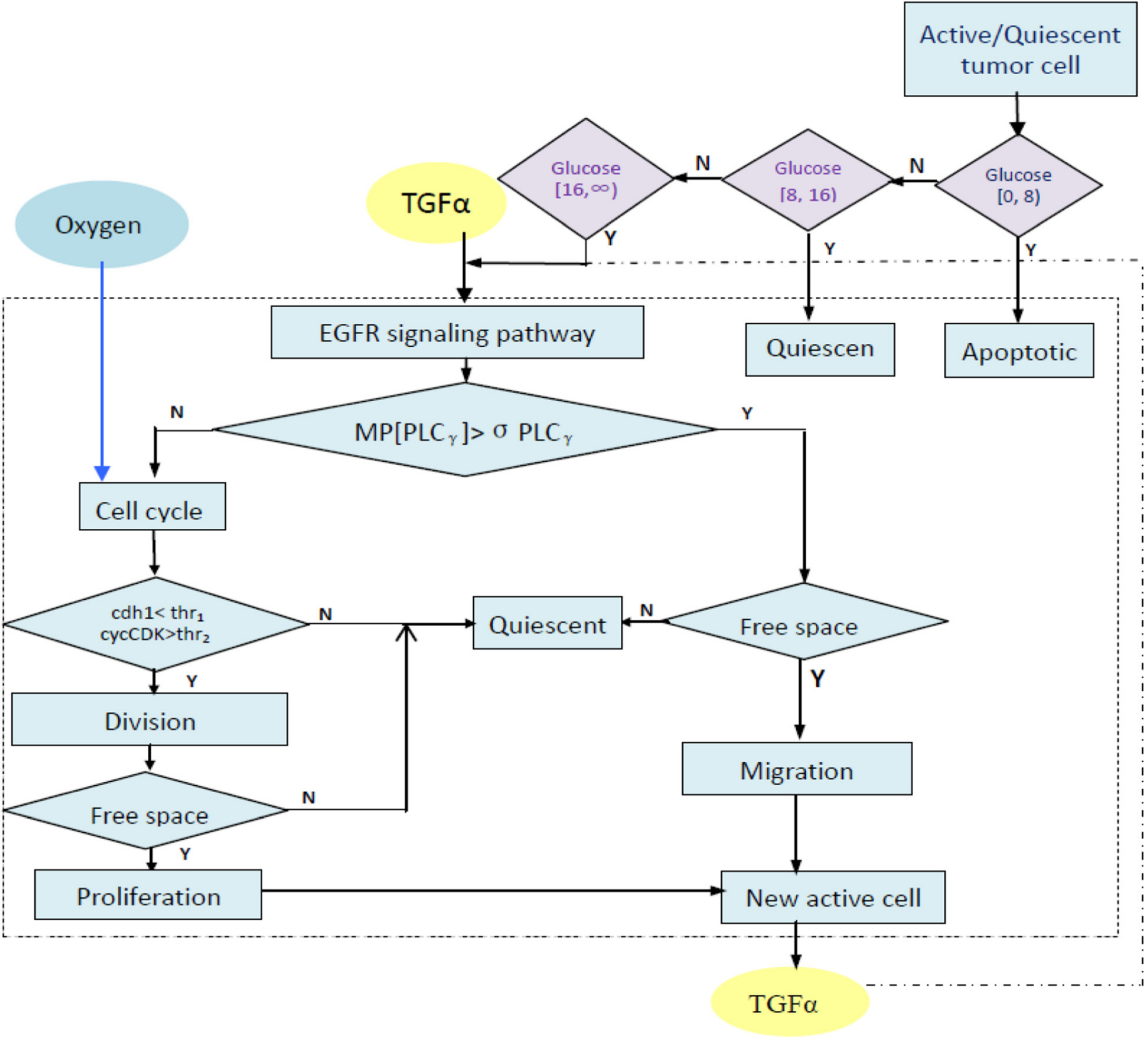
**Illustrations of phenotype switch "decision" of tumor cell.** Please see details in the main text (Cellular scale: Phenotype switch of tumor cell as "agent").

**Figure 4:**
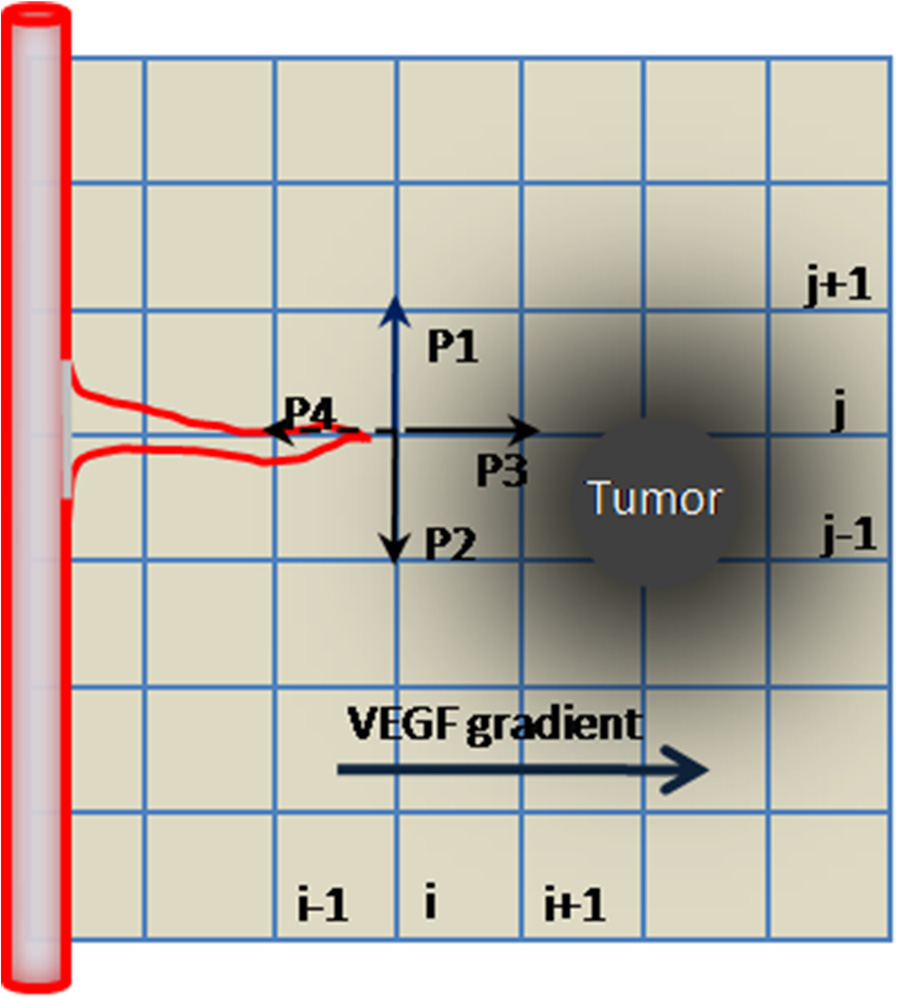
**Probability of migration of tip endothelial cell.** The tip endothelial cell probabilistically moves up, down, right, or left, or stays at its current position.

Our model encompasses four biological scales (Figure 1): the molecular scale, the cellular scale, the micro-environmental scale and the tissue scale. The molecular scale consists of the EGFR signaling and cell cycle pathways (Figure 2). The cellular scale describes the phenotypic switch of the tumor cells which is determined by the molecular scale, the cell-cell and the cell-microenvironment interactions (Figure 3). The micro-environmental scale provides a connection between the micro-vasculature (angiogenesis) and the tumor cells. Tumor cells consume glucose and oxygen nutrients. Transforming growth factor alpha (TGFα) secreted by tumor cells triggers the EGFR signaling pathway, which in turn determines cell’s phenotypic switch. Oxygen plays an important role in the cell cycle during the proliferation phase. The VEGF secreted by the tumor cells induces angiogenesis. Meanwhile, TGFα, glucose, oxygen and VEGF diffuse continuously in the tumor microenvironment. The tissue scale focuses on angiogenesis (Figure 4). Blood vessel sprouts migrate and branch via tip endothelial cells' migration in response to VEGF chemotaxis and fibronectin haptotaxis. The new blood vessels supply tumor cells with glucose and oxygen to maintain their metabolism and further invasion.

We performed our simulations on a two-dimensional square lattice. The lattice size was set to L = 200 representing a 4 ~ 5 mm length of a brain tissue slice. The lattice spacing is 20 *μm*, which is approximately the diameter of tumor cells. Initially a parent blood vessel along with 6 tip endothelial cells is located near the left boundary of the computational domain. Also a small cluster of active tumor cells were randomly distributed as shown in Figure 4. Each tumor cell is initialized with its age being between 0 ~ 24 hours randomly. The time step of the simulation is one hour. The details of the model parameters are in the Additional files 1, 2, 3, 4, and 5 of this paper.

### Molecular scale: signaling pathway

The concentration of each component (Figure 2) in the *EGFR* signaling [11,20] and cell-cycle pathways [21] are described by a system of coupled ordinary differential equations (*ODE*s) in the form

(1) $$dX_{i}/dt=\sum v_{+}-\sum v\_$$

where *v*_+_ represents the production rate of *X*_*i*_ and *v*_ the consumption rate. The parameters of *ODE*s are in Additional files 1, 2, 3, and 4. Parameter sensitivity and model robustness analysis are presented in the results section. These methods enabled us to determine which parameters of the system are more sensitive as well as to examine if the system is robust enough to the small parameter perturbations.

### Cellular scale: phenotype switch of tumor cells as "agents"

At every simulation step, each "agent" (i.e. a brain tumor cell) switches its phenotype according to the following rules:

1. First, each agent evaluates the concentration of glucose at its current location. If the concentration is greater than the cell active threshold, the agent becomes active and uses its EGFR signaling pathway to determine its phenotype. If the concentration of glucose is less than the dead threshold, the cell dies. If the concentration is between active and dead thresholds, the agent enters into a reversible quiescent state [10].

2. Each active agent evaluates its migration potential (*MP*) by the following equation:

(2) $$\text{MP}\left[\text{PLC}\gamma \right]=d\left[\text{PLC}\gamma \right]/\text{dt,}$$

where *d*[*PLCγ*]/*dt* is the rate of change of the PLCγ concentration. If *MP* is greater than a threshold σ_PLCγ,_ the average rate of change of PLCγ concentration, the agent will choose the migration phenotype.

3. If *MP* is less than σ_PLCγ_, the agent starts to proliferate. If the concentration of CDh1 is less than a threshold *thr*_1_ and the concentration of cycCDk is greater than the threshold *thr*_*2*_, the cell divides. After that, the cell chooses the most attractive free site

(detailed in equation (3)) in the neighborhood to deliver its offspring. If there is no empty neighborhood, the cell turns into a reversible quiescent state until free space becomes available.

4. Each agent chooses the "most attractive" location mentioned above according to the following probability:

(3) $$P_{j}=\psi G_{j}/F_{j}+\left(1-\psi \right)\varepsilon_{j},$$

where *Gj* is the glucose concentration at location *j, F*_*j*_ is the fibronectin concentration at *j*, *ε*_*j*_ ~N(0,1) is a normally distributed error term, the parameter ψ ∈(0, 1) represents the extent of the search precision, which is set to 0.7 [22].

### Microenvironmental scale: extracellular chemotaxis

Five extracellular micro-environmental factors, glucose, oxygen, TGFα, VEGF and fibronectin are included in this model. A set of reaction–diffusion equations describe the diffusion, penetration and uptake of glucose, oxygen and VEGF.

Glucose first penetrates blood vessels, and then diffuses in the extracellular microenvironment. After that, it is consumed by the tumor cells. This process is modeled by the following equation:

(4) $$\begin{array}{ll} \frac{\partial G}{\partial t}= & D_{G}\Delta G+X_{\text{ves}}\left(t,x\right)q_{G}\left(G^{\text{blood}},-,G\right)\\ & -X_{\text{tum}}\left(t,x\right)U_{G}, \end{array}$$

where *G* is the glucose concentration, $\Delta \equiv \nabla^{2}$ is the Laplace operator, *D*_*G*_ is the diffusivity of glucose. *q*_G_ = 2πr*p*_*G*_, where *p*_*G*_ is the vessel permeability for glucose and *r* is the blood vessels' average radius. In addition, *G*^*blood*^ is the glucose concentration in blood and *U*_G_ is the cell’s glucose uptake rate. The time dependent characteristic function *X*_*ves*_ (*t,x*) is equal to 1, if a blood vessel is present at *x*; otherwise it is equal to 0. *X*_*tum*_ (*t,x*) is equal to 1 in the tumor region and is equal to 0 elsewhere. *X*_*ves*_ and *X*_*tum*_ are updated at each simulation step according to the developing profile of the tumor and its micro-vascularity.

Oxygen also permeates the blood vessels' walls, diffuses in the surrounding and is consumed by tumor cells. This process is modeled by the following equation:

(5) $$\begin{array}{ll} \frac{\partial C}{\partial t}= & D_{C}\Delta C+X_{\text{ves}}\left(t,x\right)q_{C}\left(C^{\text{blood}}-C\right)\\ & -X_{\text{tum}}\left(t,x\right)U_{C}, \end{array}$$

where *C* is the oxygen concentration, *D*_*C*_ is the oxygen diffusivity, *q*_*C*_ is the vessel permeability for oxygen, and *U*_*C*_ is a cell’s uptake rate of oxygen.

TGFα, an analogue of EGF, is secreted by tumor cells and can be paracrine and juxtacrine [23]. Equation (6) describes the diffusion and secretion of TGFα.

(6) $$\begin{array}{ll} \frac{\partial T}{\partial t}= & D_{T}\Delta T-X_{\text{ves}}\left(t,x\right)q_{T}T+X_{\text{tum}}\left(t,x\right)S_{T}\\ & -\delta_{T}T, \end{array}$$

where *T* is the TGFα concentration*, D*_*T*_ is its diffusivity, *q*_*T*_ is vessel permeability to TGFα. *S*_*T*_ is a cell’s net production rate of TGFα and *δ*_*T*_ is the natural decay rate of TGFα.

We applied homogeneous Neumann boundary conditions for all the above equations by assuming zero flux along the boundary of the considered domain. Additional file 5: Table A5 and Additional file 6: Equations A1–A5 list the parameters and initial conditions of the equations**.** We solved these equations numerically with the finite difference method [24].

### Tissue scale: angiogenesis

Tumor induced angiogenesis is due to the secretion of VEGF by the tumor cells. VEGF diffuses into the surrounding corneal tissue and is also consumed by the endothelial cells [25]. We model this process with the following equation:

(7) $$\begin{array}{ll} \frac{\partial V}{\partial t}= & D_{V}\Delta V-X_{\text{ves}}\left(t,x\right)q_{V}V+X_{\text{tum}}\left(t,x\right)S_{V}\\ & -\delta_{V}V, \end{array}$$

where *V* is the VEGF concentration, *D*_*V*_ is the diffusivity of VEGF, *q*_*V*_ is the vessel permeability for VEGF and *S*_*V*_ is a cell’s VEGF secretion rate. *δ*_*V*_ is the natural decay rate of VEGF.

Fibronectin is a component of the corneal tissue secreted by endothelial cells. In addition, tumor cells can consume fibronectin. This process is described by the following equation:

(8) $$\frac{\partial F}{\partial t}=X_{\mathrm{ves}}\left(t,x\right)\beta -X_{\mathrm{tum}}\left(t,x\right)\gamma F,$$

where *F* is the fibronectin concentration, β and γ are positive constants representing the production and uptake rates, respectively.

We assume that the motion of individual endothelial cell (EC) located at the tip of a capillary sprout governs the motion of the whole sprout. Chemotaxis in response to VEGF gradients and haptotaxis in response to fibronectin are the major factors that influence the motion of the endothelial cells at the capillary sprout tip [25].

We defined the probability of migration of a tip endothelial cell (see Figure 4) as

(9) $$\begin{array}{l} P_{k}\propto \left(\alpha \frac{k_{v}}{k_{v}+V}\nabla V+\lambda \nabla F\right).l_{k},\cdots \cdots k\\ \;=1,2,3,4, \end{array}$$

where *V* is the VEGF concentration, *F* is fibronection concentration and *l*_*k*_ is the directional vector along the *k*th direction. The term $\alpha k_{v}/\left(k_{v}+V\right)$ ∇*V* models chemotaxis in response to VEGF gradients [19], where α is the chemotactic coefficient and *k*_*v*_ is a positive constant controlling the weight of VEGF concentration in chemotactic sensitivity. The term *λ*∇*F* models the haptotatic influence of fibronectin on the endothelial cells, where *λ* is the haptotatic coefficient.

For a given tip of endothelial cells at location (*i, j*), the un-normalized migration probabilities can be calculated from equation (9) as follows:

(10) $$\begin{array}{ll} P_{1}= & \alpha k_{V}/\left(k_{V}+V\left(i,j\right))\cdot (V\left(i,j+1\right)-V\left(i,j\right)\right)\\ & +\lambda (F(i,j+1)-F(i,j)),\\ P_{2}= & \alpha k_{V}/\left(k_{V}+V\left(i,j\right))\cdot (V\left(i,j-1\right)-V\left(i,j\right)\right)\\ & +\lambda (F(i,j-1)-F(i,j)),\\ P_{3}= & \alpha k_{V}/\left(k_{V}+V\left(i,j\right))\cdot (V\left(i+1,j\right)-V\left(i,j\right)\right)\\ & +\lambda (F(i+1,j)-F(i,j)),\\ P_{4}= & \alpha k_{V}/\left(k_{V}+V\left(i,j\right))\cdot (V\left(i-1,j\right)-V\left(i,j\right)\right)\\ & +\lambda (F(i-1,j)-F(i,j))\text{.} \end{array}$$

The un-normalized probability *P*_*5*_, for a tip cell to remain stationary is the average of *P*_1_, *P*_2_*, P*_3_ and *P*_4._ After normalization, the above equations give the likelihood of the tip endothelial cell to move up, down, right, or left, or stay at its current position. The probability, *P*_*5*_, for a tip cell to remain stationary is the average of *P*_1_, *P*_2_*, P*_3_ and *P*_4_.

The algorithm related to angiogenesis is as follows:

1. Calculate the migration probabilities of ECs:

1.1 Solve the equations (7) and (8), and then calculate *P*_1_*-P*_5_ from (10);

1.2 Normalize the above numbers: ${\tilde{P}}_{i}=P_{i}/\sum_{j=1}^{5}P_{j},.i=1,2,\ldots ,5$; define intervals $I_{1}=(0,{\tilde{P}}_{1}],I_{i}=(\sum_{j=1}^{i-1}{\tilde{P}}_{j},\sum_{j=1}^{i}{\tilde{P}}_{j}],i=2,\ldots ,5$.

2. For every sprout tip cell, we check whether the age of vessel is greater than 18 hours and whether there are any free sites in its nearest neighborhood.

2.1 Sprout branching: If the above conditions are satisfied, two random numbers *r*_1_ and *r*_2_ between 0 and 1 are generated. If *r*_1_ ∈ *I*_2_ and *r*_2_ ∈ *I*_3_, then we move two endothelial cells one below and one to the right of the spout tip endothelial cell.

2.2. Sprout migrating: If the above branching conditions are not satisfied, we generate another random number *r* between 0 and 1. If *r* ∈ *I*_3_, we move the tip endothelial cell to the right of spout tip endothelial cell.

3. Anastomosis: If two sprouts encounter each other, a new sprout continues to grow.

### TKI treatment

This study uses TKIs, particularly gefitinib, as inhibitors delivered by capillary vessels to treat brain cancer. The TKIs are modeled as a continuous concentration field. These molecules permeate the blood vessels and diffuse continuously in the microenvironment to produce an accumulative inhibitive effect on the tumor growth. We describe the evolution of the concentration of TKIs by the following equation:

(11) $$\begin{array}{l} \frac{\partial Tki}{\partial t}=D_{\mathrm{Tki}}\Delta Tki\\ \;+X_{\mathrm{ves}}\left(t,x\right)q_{\mathrm{Tki}}\left(Tki^{\mathrm{blood}}-Tki\right)\\ \;-X_{\mathrm{tum}}\left(t,x\right)U_{\mathrm{Tki}}-\delta_{\mathrm{Tki}}Tki, \end{array}$$

where *D*_*TKi*_ is the diffusivity of the TKIs, *q*_*TKi*_ is the vessel permeability for TKIs, *TKi*^*blood*^ is the blood TKIs concentration, *U*_*TKi*_ is a cell’s uptake rate of TKIs, and *δ*_*Tki*_ is the natural decay rate of TKIs.

The EGFR signaling pathway controls a cell’s phenotypic switch by binding TGFα molecules to the EGF receptors. Because the TKI molecules inhibit the autophosphorylation receptors, the downstream EGFR pathway responsible for a cell's phenotypic switch remains inactivated [26]. The binding and unbinding processes of TKIs to EGFR with constant rates *k*_*b*_ and *k*_*u*_, respectively, are described by the following equation:

(12) $$EGFR+TKI\overset{k_{b}}{\underset{k_{u}}{⇄}}EGFR:TKI$$

Since the chemical reaction rate is much faster than the cells' phenotypic switch [26], the concentration of EGFR: TKI complex in equation (12) can be obtained by applying Michaelis–Menten kinetics as follows:

(13) $$\left[EGFR:TKI\right]=\frac{{\left[EGFR\right]}_{0}\left[TKI\right]}{k_{m}+\left[TKI\right]}$$

where *k*_*m*_ is the Michaelis constant, *k*_*m*_ ≈ *k*_*b*_ / *k*_*u*_, and [EGFR]_0_ is the initial concentration of the EGFR. We can then derive the effective amount of EGFR for the activation of downstream factors as follows:

(14) $${\left[EGFR\right]}_{\mathrm{eff}}={\left[EGFR\right]}_{0}-\left[EGFR:TKI\right]$$

Because of the TKI treatment, the effective amount of EGFR of some tumor cells will decrease. The decrease of the amount of effective EGFR results in a slow rate of change of PLCγ concentration. This in turn inhibits tumor progression by reducing the migration potential of these tumor cells (see equation 2 and detailed phenotype change of tumor cells in Figure 3).

Finally, we summarize our computing algorithm at each step across multi-scales (Figure 1) as follows. At micro-environmental scale, we solve the PDEs (equations 4–6) to obtain the spatial concentration distributions of glucose, oxygen and TGFα. At molecular scale, we use the calculated local TGFα concentration as the input for EGFR signaling pathway (equation 1) for each tumor cell. At cellular scale, tumor cells' migration potential (MP) (equation 2) is computed to determine their phenotypic switch (migration or proliferation); meanwhile, other phenotypic switches (quiescent or apoptosis) are associated with the current value of oxygen and glucose. At tissue scale, the spatial concentration distributions of VEGF and fibronectin (equations 7–8) will guide the tip endothelial cells' migration and sprout branching. In turn, the remodeled vasculature at tissue scale influences the spatial concentration distributions of glucose, oxygen and TGFα at micro-environmental scale. For TKI treatment, the TKI distribution is integrated into molecular scale by solving equation 11 along with aforementioned equations 4–6, and the initial value of EGFR is varied by equations 12–14 as well.

## Results

We have implemented the above model into software "ABM-TKI" in the Matlab programming environment. “ABM-TKI” is a tool employing agent-based model (ABM) to simulate brain tumor growth. It includes an EGFR signaling pathway, a related cell-cycle, angiogenesis and TKIs treatment. We can employ this tool to predict the responses of brain cancer and reveal the dual roles of angiogenesis under TKI treatment.

Regarding software usage, the user can download and decompress the package from the project home page (https://sites.google.com/site/agentbasedtumormodeling/home). Then the user can run the program in Matlab (version R2007b or higher) with input as: angiog_tumor(time,isdrug).The input "time" is the period from the beginning of the simulation to the end. The "isdrug = 1" means TKI treatment and "isdrug = 0" means no TKI treatment. For example: "angiog_tumor(150,0)" will give the tumor growth profile without TKI treatment from 0 hour to 150 hours; "angiog_tumor(300,1)" will give the tumor growth profile with TKI treatment from 0 hour to 300 hours.

The output includes: (a) the vascular tumor growth pattern with or without TKI treatment; (b) the tumor growth visualization with the background of fibronectin; (c) the spatio-temporal evolution of the concentration of glucose, oxygen, TGFα and/or TKI; (d) various tumor cell numbers such as active cells, apoptotic cells, migratory cells, proliferative cells, quiescent cells and the number of endothelial cells; (e) the average change rate of PLCγ with or without TKI treatment.

### Vascular tumor growth patterns

**Figure 5:**
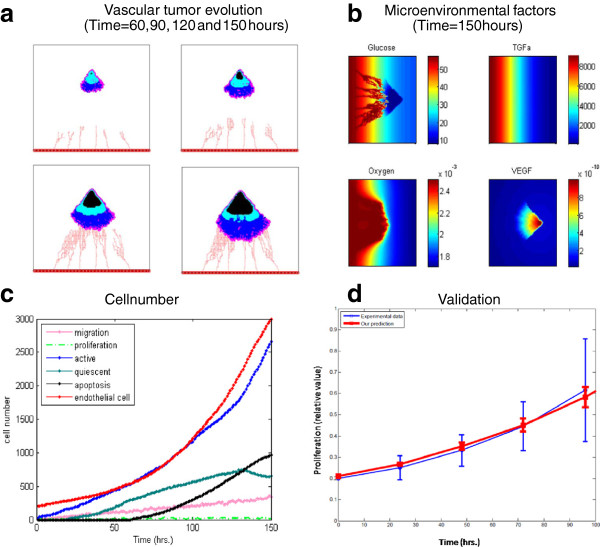
**Simulation of vascular tumor growth without TKI treatment.** (**a**) The evolution of vascular tumor patterns at 60, 90, 120 and 150 hours. (**b**) The distribution of glucose, oxygen, TGFα and VEGF in microenvironment at 150 hours. (**c**) Different types of cell numbers from 0 hour to 150 hours. (**d**) Proliferation rate of tumor cells derived from simulations (**red line)** and from *in vitro* experimental observations (**blue line**) for t=0-96 hours.

The vascularized tumor patterns at 60, 90, 120 and 150 hours are shown in Figure 5a, where different colors denote various tumor cell states: active (blue), quiescent (cyan), dead (black) and proliferative (pink). The endothelial cells are red. (See also Additional file 7: Figure A1 for more details). The active tumor cells are comprised of migratory cells, cells that have just completed their proliferation process, and cells that just switched back from quiescent state to the active state. Additional file 8: Figure A2 shows the growth of the tumor microvascularity and the fibronectin concentration at different time interval. Figure 5a showed that those tumor cells tend to migrate to locations near the vessels, where the glucose concentration level is the highest. Around t = 60 hours, the tumor was comprised of active cells and some quiescent cells. Around t = 90 hours some dead cells appeared in the tumor mass. From t = 120 hours to t = 150 hours, the number of both dead and active cells kept increasing. At t = 150 hours, we found that some quiescent cells near blood vessels switched back to active cells. At this time, the tumor developed a tree branching microvasculature, which is much denser near the tumor. Figure 5b shows the distributions of different micro-environmental factors (glucose, TGFα, oxygen, VEGF) at t = 150 hours. Given the space limitation, we show the results at a single time interval. The concentrations of these molecules at different times are shown in the Additional file 9: Figure A3, with different colors representing different levels of concentration. We found that the descending order of the diffusion rate for the four molecular species is as follows: oxygen, glucose, VEGF or TGFα.

Figure 5c shows the numbers of different types of cells as a function of time. The number of active cells increased monotonically with time. The number of apoptotic cells increased abruptly at around t = 60 hours and kept increasing until the tumor microvasculature developed at t = 150 hours. The number of quiescent tumor cells kept increasing from t = 0 to 130 hours, and then began to decrease. The number of endothelial cells increased rapidly during the whole simulation time. The detailed evolutions of the numbers of various cells are shown in Additional file 10: Figure A4 separately.

In Figure 5d, we present the proliferation rate of tumor cells as a function of time from our simulation and from *in vitro* experimental results (at t = 96 hours) [27]. The *in vitro* experimental data are from human glioma tumor-initiating cells derived from 7 patients (GBM 1–7). The plotted experimental data are the mean and standard deviation values from the seven cell lines. We took the mean value of these data as the blue line in Figure 5d. The mean squared error of our prediction is 0.1421. The simulation and experimental data in Figure 5d are in very good agreement, which is an important validation of our model.

### TKI treatment response

**Figure 6:**
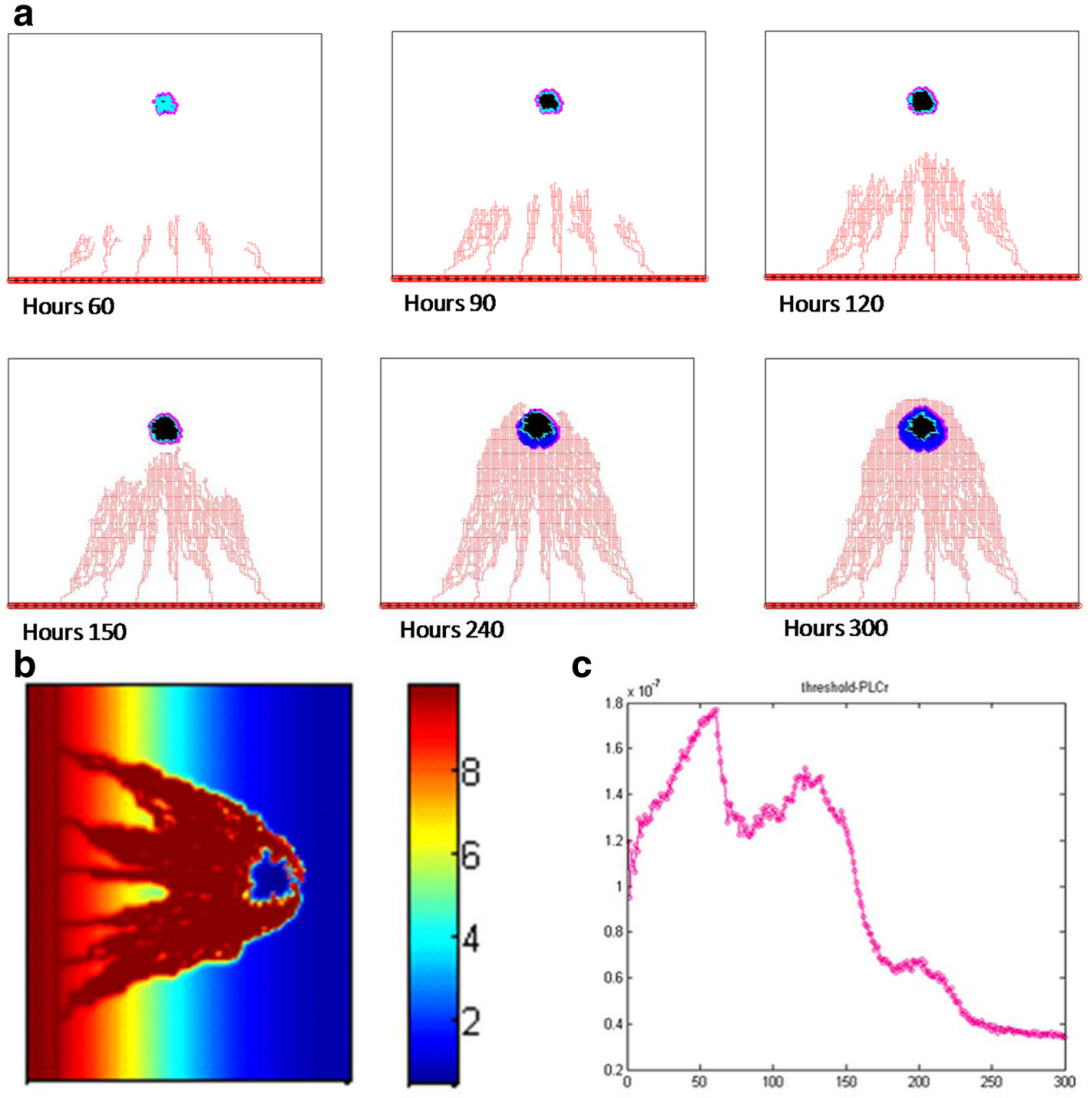
**Simulation of vascular tumor growth with TKI treatment.** (**a**) Vascular tumor growth patterns with TKI treatment at 60, 150, 240 and 300 hours; (**b**) The distribution of TKI in tumor vasculature at 300 hours; (**c**) Average change rate of PLCγ from hour 0 to 300 hours.

In our study we chose gefitinib as the EGFR TKI to treat brain cancer. In this section the term TKI refers to treating cell by gefitinib. Figure 6a shows the simulation profiles of the vascularized tumor growth with TKI treatment from t = 0 to 300 hours. The different colors represent cell types as defined in Figure 5a. The results in Figure 6a show that the tumor expansion slowed down obviously by the TKI treatment. Additional files 11- 12: Figure A5-6 show the detailed vascular tumor growth profile with TKI treatment as well as that in the presence of fibronectin at different time intervals.

Figure 6b shows the distribution profile of TKIs concentration at t = 300 hours which is similar to the structure of tumor microvasculature. The Additional file 13: Figure A7 and Additional file 14: Figure A8 demonstrate the evolution of distributions of glucose, oxygen, TGFα and VEGF as well as TKIs during the treatment at different time intervals.

Figure 6c shows the average rate of change of PLCγ as a function of time. The PLCγ average rate of change increases from t = 0 to 60 hours and then it starts decreasing with an exception at around t = 75 hours where the data show a hump. Finally, the curve goes down after t = 125 hours. Since Additional file 10: Figure A4 shows the average rate of change of PLCγ always increases without TKI treatment, TKI treatment greatly affects the average rate of change of PLCγ. The numbers of various cells with TKI treatment are shown in Additional file 15: Figure A9.

**Figure 7:**
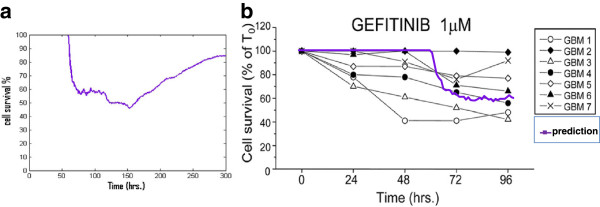
**Prediction and validation of cell survival percentage during TKI treatment.** (**a**) Prediction of cell survival percentage during TKI treatment from 0 hour to 300 hours; (**b**) Cell survival percentage from simulations (**purple line**) and from in vitro experimental observations (**black line**) for t=0-96 hours.

Figure 7a shows the cell survival rate under TKI treatment from t = 0 to 300 hours which decreased until t = 150 hours and then increased again until t = 300 hours.

Figure 7b demonstrates that the simulated cell survival rate has a trend similar with experimental results. The purple line in the figure represents the average from a hundred simulations. Human glioma tumor-initiating cells are derived from 7 patients (GBM 1–7) [27] for *in vitro* experiments observed for 96 hours, these experimental data are shown by the multiple lines in the figure. In the experiment human glioma tumor growth inhibitors of gefitinib are used as TKI at the concentration of 1 μM, while in our simulation we also chose gefitinib as TKI and its concentration near tumor region is also close to 1 μM from t = 0 to 100 hours. The relatively good agreement between the simulation prediction and the experimental results constitutes an important validation of our model.

### Sensitivity analysis and model robustness

Parameter sensitivity analysis is to quantitatively discover sensitive parameters in the system. And robustness analysis is to examine whether the system is stable to modest fluctuations of these sensitive parameter values.

**Figure 8:**
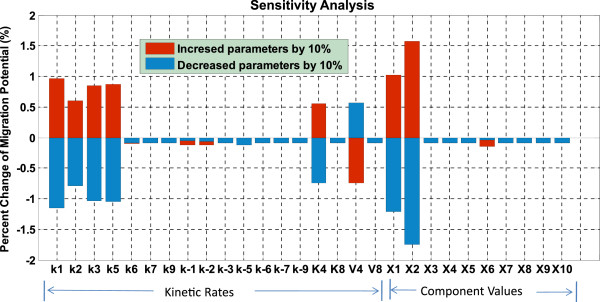
**Sensitivity analysis of EGFR signaling pathway.** The result shows that the migration potential of brain cancer cells is most sensitive to the initial concentration of EGFR (*X*_2_) and TGFα (*X*_1_). The result also shows us forward kinetic rates (*k*_1_, *k*_2_, *k*_3_, *k*_5_) are more important in the system and demonstrate that the developed intracellular pathway system is robust.

In this work, local parameter sensitivity analysis [28-30] was employed to understand the relationship between the migration potential (defined above) of tumor cell and the variations in individual parameter values. The sensitivity coefficient (S) is calculated according to the following formula [31]:

(15) $$S=\frac{\Delta MP}{MP}/\frac{\Delta P}{P},$$

where P is the parameter that is varied and MP is tumor cell's migration potential; Δ*MP* is the corresponding change in MP due to a small change in P denoted by Δ*P*. Each individual parameter is increased (or decreased) by 10% from its original value, and the resulting percentage change of the migration potential is computed. Figure 8 shows that the migration potential of the cells is most sensitive to the initial concentration of EGFR (*X*_2_) and TGFα (*X*_1_). The sensitivity analysis also demonstrates that kinetic rates, *k*_1_, *k*_2_, *k*_3_, *k*_5_, *K*_4_ and *V*_4_, are more critical than others in the system. Furthermore, it turns out that the developed intracellular pathway system is rather robust since all of the sensitivity coefficients are less than 1.8%.

Based on above sensitivity analysis results, we selected 3 pathway components, TGFα (*X*_1_), EGFR (*X*_2_) and PLCγ (*X*_6_), and 6 kinetic rates including *k*_1_, *k*_2_, *k*_3_, *k*_5_, *K*_4_ and *V*_4_ as key parameters to investigate the robustness of the model to much larger ranges of parameter variations. For each of these parameters, we varied its value by 0.1-fold, 2.0 fold, 5.0 fold, 10.0 fold, 50.0-fold, and 100.0-fold of its original reference value respectively. Each time, only one of the above parameters was varied with a corresponding fold change, and all other parameters were kept fixed. We chose active cell number (*ACN*) at 100 hours in each simulation as the system outcome, and examined the system responses to the above parameter variations. We defined the robustness index (R) as follows:

(16) $$R=\frac{ACNp-ACN0}{ACN0},$$

where *ACN*_*p*_ is the active cell number with the varied parameter *p*, and *ACN*_*0*_ is the average active cell number from the 100 simulations with unvaried reference parameters.

**Figure 9:**
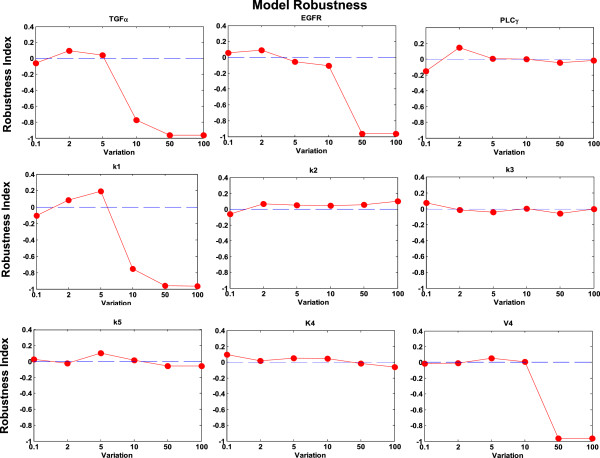
**Model robustness analysis.** TGFα (*X*_1_), EGFR (*X*_2_), PLCγ (*X*_6_), and *k*_1_, *k*_2_, *k*_3_, *k*_5_, *K*_4_ and *V*_4_ were varied their values by 0.1-fold, 2.0 fold, 5.0 fold, 10.0 fold, 50.0-fold, and 100.0-fold of original reference values each time. The results show that the absolute values of the robustness indexes are less than 0.1894 with respect to most parameters (except TGFα and *k*_1_) for variations between 0.1-fold and 10.0-fold.

**Figure 10:**
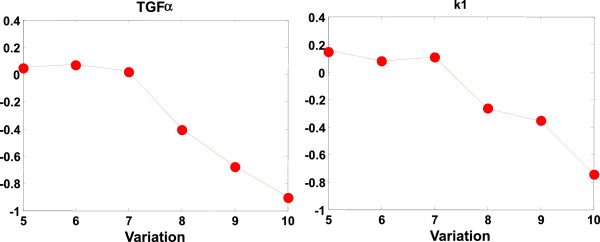
**Detailed robustness analyses for TGFα and *k***_**1**_**.** TGFα (*X*_1_), and *k*_1_ were varied by 5.0-fold, 6.0 fold, 7.0 fold, 8.0 fold, 9.0-fold, and 10.0-fold of original reference values each time. The results show the absolute values of the robustness indexes are less than 0.1894 with respect to TGFα and *k*_1_ for variations between 0.1-fold and 7.0-fold.

Figure 9 shows that for variations between 0.1-fold and 10.0-fold, the absolute values of the robustness index is less than 0.1894 with respect to most parameters (except TGFα and *k*_1_). Figure 10 shows the detailed analyses of the system robustness with respect to TGFα and *k*_1_ at variations between 5.0-fold to 10.0-fold. The results show that regarding TGFα and *k*_1_, at the variations between 0.1-fold and 7.0-fold, the absolute values of the robustness indexes are less than 0.1894. Furthermore, for some parameters, such as PLCγ, *k*_2_, *k*_3_, *k*_5_, *K*_4_, the robustness index is very small even with the variations up to 100.0-fold. Then we calculated the coefficient of variation (CV) of system outcomes which is defined as the ratio of the standard deviation to the mean of the above variational active cell numbers. At the parameter variations between 0.1-fold and 10.0-fold, the CV of system outcomes is 0.2007 which is much less than 1, indicating that the system is relatively stable to the parameter variations. These results reveal that our model is comparably robust with respect to relatively large ranges of parameter variations.

## Discussion

We developed a multi-scale model by integrating a novel angiogenesis module into an agent-based tumor model based on a set of reaction–diffusion equations that describe the spatio-temporal evolution of the distributions of micro-environmental factors such as glucose, oxygen, TGFα, VEGF and fibronectin. These molecular species regulate tumor growth during angiogenesis. Each tumor cell is equipped with an EGFR signaling pathway linked to a cell-cycle to determine its phenotype.

Our simulations show several interesting findings. The first is that tumor cells tend to move towards blood vessels and gradually developed to a fan-shape as shown in Figure 5a. We interpret this result as follows. Since the nutrients (glucose and oxygen) concentrations are higher at locations near the blood vessels, they attract tumor cells.

The second interesting finding is that blood vessels tend to migrate to tumor and form a dense tree-branching vascular network. The reason is that a high VEGF gradient close to the tumor attracts endothelial cells, which in turn lead to branching of vessels in these regions.

The third interesting finding is that TKI treatment can inhibit tumor progression. The binding of TKI molecules to EGFR decreases the amount of effective EGFR, which results in low expression of PLCγ and low cell’s migration potential (Figure 6c). As a result, tumor invasion slows down.

The fourth interesting result is that the tumor cells' survival rate does not always decrease. This is due to the dual role of angiogenesis. Newly formed capillaries delivers a substantial amount of TKI molecules to tumor cells and blocks the EGFR signaling pathway, which lead to an inhibition of tumor growth. This in turn results in a decreased cell survival rate in the early stage of the tumor development. On the other hand, new capillaries transported a lot of glucose and oxygen to tumor cells which results in an increased cell survival rate at later stages (Figure 7a). The implications of the dual roles of angiogenesis reveal that clinical personnel should decrease cancer progression by using TKI treatment and inhibiting tumor-induced angiogenesis at the same time.

The sensitivity analysis reveals sensitive parameters in the EGFR signaling pathway. The robustness study confirms that our model is relatively robust and stable to fluctuations of these sensitive parameters.

Herein we used the two-dimensional *in vitro* experiments [27] to validate the effectiveness of the model. These experiments employed two-dimensional experimental protocol to isolate and plate human glioma tumor-initiating cells in Martrigel-coated culture flasks. Figure 5d and Figure 7b demonstrate that our *in silico* model does have strong predictive power and great potential for clinical work.

We are going to extend the model to three dimensions to simulate *in vivo* tumor growth for real clinical purposes. A three-dimensional lattice is indispensable for the simulation of *in vivo* tumor growth with angiogenesis, because tumor cells' activities, vasculature structure, and chemical cues' diffusion in three-dimensional heterogeneous tumor growth environment are very different from two-dimensional. Moreover, three-dimensional simulations require parallel computing techniques [32] to relieve the heavy computing request.

The potential of our model will further increase, by incorporating more realistic biological and physical features, such as blood flow and tumor growth-induced pressure [33] in the future.

## Conclusions

This work presents a novel multi-scale agent-based brain tumor model encompassing an EGFR signaling pathway together with a related cell-cycle, an angiogenesis module and TKI treatment. It incorporates four relevant biological scales: the molecular scale, the cellular scale, the microenvironment scale and the tissue scale. At the molecular scale, a system of ordinary differential equations simulates the dynamics of the EGFR signaling pathway and the cell cycle to determine the cells' phenotypic switch at the cellular scale. We employed a set of partial differential equations to simulate the concentration changes of five extracellular chemical cues (glucose, oxygen, TGFα, VEGF and fibronectin) in the tumor micro-environmental scale. Angiogenesis was coupled into tumor growth through VEGF secreted by the tumor cells and through the glucose and oxygen permeated from the neo-vasculature at the tissue scale. Moreover, we integrated TKI treatment into EGFR signaling pathway to block the activation of EGFR.

Our simulations demonstrate that the entire tumor growth profile is a collective behaviour of its cells regulated by the EGFR signaling pathway and the cell cycle. We also discovered that angiogenesis has dual effects on TKI treatment: on one hand, neo-vasculature can deliver TKIs to decrease the tumor invasion, whereas on the other hand, it can transport a lot of nutrients ( glucose and oxygen) to tumor cells to maintain their metabolism, which results in an increase of cell survival rate at late simulation stage. There is a great similarity between the simulation results and existing *in vitro* experimental data. Further analyses show that our model has strong robustness regarding to the relatively large changes of the sensitive model parameters.

## Availability and requirements

**Project name:** multi-scale agent-based brain tumor modeling project **Project home page: **http://www.methodisthealth.com/Softwarehttp://csysbio.org/Released%20Software.htmlhttps://sites.google.com/site/agentbasedtumormodeling/home**Operating system(s):** Platform independent **Programming language:** Matlab (R2007b) **Other requirements:** None **License:** GNU GPL, FreeBSD etc. **Any restrictions to use by non-academics:** license needed.

## Abbreviations

EGFR: Epidermal Growth Factor Receptor; VEGF: Vascular Endothelial Growth Factor; TKIs: Tyrosine Kinase Inhibitors; EC: Endothelial Cell; MP: Migration Potential.

## Competing interests

The authors declare that they have no competing interests.

## Authors' contributions

XS participated to study conception, carried out the model programming, carried out the analysis of the model and drafted the manuscript. XZ participated to study conception and improved the manuscript. LZ participated to study conception, model analysis and improved the manuscript. HT participated to study conception and helped to initiate the model programming. JB helped to study conception. CS helped to improve the manuscript. All authors read and approved the final manuscript.

## Supplementary Material

Additional file 1 **Table A1.** Kinetic equations describing the reactions between the components of the simplified EGFR signaling pathway. Click here for file

Additional file 2 **Table A2.** Coefficients of the simplified EGFR signaling pathway. Click here for file

Additional file 3 **Table A3.** Kinetic equations describing the reactions between the components of the cell-cycle. Click here for file

Additional file 4 **Table A4.** Parameter in cell-cycle pathway. Click here for file

Additional file 5 **Table A5.** Parameters of microenvironmental PDEs in the model. Click here for file

Additional file 6 **Text A1.** Equations describing the Initial distribution of glucose, oxygen, TGFα, VEGF and fibronectin. Click here for file

Additional file 7 **Figure A1.** Tumor induced angiogenesis and vascular tumor growth without TKI treatment. Click here for file

Additional file 8 **Figure A2.** Vascular tumor growths with concentration change of fibronectin. Click here for file

Additional file 9 **Figure A3.** The concentration change of glucose, oxygen, TGFα and VEGF without TKI treatment. Click here for file

Additional file 10 **Figure A4.** Various tumor cell numbers without TKI treatment. Click here for file

Additional file 11 **Figure A5.** Vascular tumor growth with TKI treatment. Click here for file

Additional file 12 **Figure A6.** Vascular tumor growth in the presence of fibronectin with TKIs treatment. Click here for file

Additional file 13 **Figure A7.** The concentration change of glucose, oxygen, TGFα and VEGF with TKI treatment. Click here for file

Additional file 14 **Figure A8.** The concentration change of TKIs shown at 60, 150, 240 and 300 hours. Click here for file

Additional file 15 **Figure A9.** Various tumor cell numbers with TKI treatment. Click here for file
