# Supplementary material for: Multi-scale agent-based brain cancer modeling and prediction of TKI treatment response: Incorporating EGFR signaling pathway and angiogenesis
Source: BMC Bioinformatics. 2012 Aug 30;13:218. doi: 10.1186/1471-2105-13-218 (PMC3487967; doi:10.1186/1471-2105-13-218)
Supplement: Additional file 1 — Table A1. Kinetic equations describing the reactions between the components of the simplified EGFR signaling pathway. [file 1471-2105-13-218-S1.doc]

**Table 1** Kinetic equations describing the reactions between the components of the simplified EGFR signaling pathway; the initial values of the various variables are taken from [10, 20]. The initial value of the effective EGFR is varied in the simulation with TKI treatment (see main text).

| **Symbol** | **Molecular variables** | **Kinetic equations** | **Initial values**  ($nM$) |
| --- | --- | --- | --- |
| $X_{1}$ | $TGF_{\alpha}$ | $dX_{1}/dt=-k_{1}X_{1}X_{2}+k_{-1}X_{3}$ | **To be Varied** |
| $X{}_{2}$ | $EGFR$ | $dX_{2}/dt=-k_{1}X_{1}X_{2}+k_{-1}X_{3}$ | 100 |
| $X{}_{3}$ | $TGF_{\alpha}-EGFR$ | $dX_{3}/dt=k_{1}X_{1}X_{2}-k_{-1}X_{3}-2k_{2}X_{3}X_{3}+2k_{-2}X_{4}$ | 0 |
| $X{}_{4}$ | $(TGF_{\alpha}-EGFR)_{2}$ | $dX_{4}/dt=k_{2}X_{3}X_{3}-k_{-2}X_{4}+V_{4}X_{5}/(K_{4}+X_{5})-k_{3}X_{4}-k_{-3}X_{5}$ | 0 |
| $X{}_{5}$ | $TGF_{\alpha}-EGFR-P$ | $dX_{5}/dt=k_{3}X_{4}-k_{-3}X_{5}+k_{7}X_{8}-k_{-7}X_{5}X_{9}-V_{4}X_{5}/(k_{4}+X_{5})-k_{5}X_{5}X_{6}+k_{-5}X_{7}$ | 0 |
| $X{}_{6}$ | $PLC_{\gamma}$ | $dX_{6}/dt=V_{8}X_{9}/(K_{8}+X_{9})-k_{5}X_{5}X_{6}-k_{-5}X_{7}$ | 10 |
| $X{}_{7}$ | $TGF_{\alpha}-EGFR-PLC_{\gamma}$ | $dX_{7}/dt=k_{5}X_{5}X_{6}-k_{-5}X_{7}-k_{-6}X_{8}$ | 0 |
| $X{}_{8}$ | $TGF_{\alpha}-EGFR-PLC_{\gamma}-P$ | $dX_{8}/dt=k_{6}X_{7}-k_{-6}X_{8}-k_{7}X_{8}+k_{-7}X_{5}X_{9}$ | 0 |
| $X{}_{9}$ | $PLC_{\gamma}-P$ | $dX_{9}/dt=k_{7}X_{8}-k_{-7}X_{5}X_{9}-V_{8}X_{9}/(K_{8}+X_{9})$ | 0 |
| $X{}_{10}$ | $PLC_{\gamma}-P-I$ | $dX_{10}/dt=k_{9}X_{9}-k_{-9}X_{10}$ | 0 |
