# Supplementary material for: Multi-scale agent-based brain cancer modeling and prediction of TKI treatment response: Incorporating EGFR signaling pathway and angiogenesis
Source: BMC Bioinformatics. 2012 Aug 30;13:218. doi: 10.1186/1471-2105-13-218 (PMC3487967; doi:10.1186/1471-2105-13-218)
Supplement: Additional file 2 — Table A2. Coefficients of the simplified EGFR signaling pathway. [file 1471-2105-13-218-S2.doc]

**Table 2** Coefficients of the simplified EGFR signaling pathway taken from [11, 20].

| **Forward rate**($s^{-1}$) | **Reverse rate**($s^{-1}$) | **Michaelis constants**($nM$) | **Maximal enzyme rates**($nMs^{-1}$) |
| --- | --- | --- | --- |
| $k_{1}=0.003$ | $k_{-1}=0.06$ | $K_{4}=50$ | $V_{4}=450$ |
| $k_{2}=0.01$ | $k_{-2}=0.1$ | $K_{8}=100$ | $V_{8}=1$ |
| $k_{3}=1$ | $k_{-3}=0.01$ |  |  |
| $k_{5}=0.06$ | $k_{-5}=0.2$ |  |  |
| $k_{6}=1$ | $k_{-6}=0.05$ |  |  |
| $k_{7}=0.3$ | $k_{-7}=0.006$ |  |  |
| $k_{9}=1$ | $k_{-9}=0.03$ |  |  |
