# Supplementary material for: Multi-scale agent-based brain cancer modeling and prediction of TKI treatment response: Incorporating EGFR signaling pathway and angiogenesis
Source: BMC Bioinformatics. 2012 Aug 30;13:218. doi: 10.1186/1471-2105-13-218 (PMC3487967; doi:10.1186/1471-2105-13-218)
Supplement: Additional file 3 — Table A3. Kinetic equations describing the reactions between the components of the cell-cycle. [file 1471-2105-13-218-S3.doc]

**Table 3** Kinetic equations describing the reactions between the components of the cell-cycle pathway. The equation for Protein 27 (*X14*) in the cell cycle of a tumor cell is taken from [21], the other equations are taken from [10].

| **Symbol** | | **Molecular variables** | **Kinetic equations** | **Initial values**($DC$) |
| --- | --- | --- | --- | --- |
| $X_{11}$ | Cdh1-APC complex | | $dX_{11}/dt$ $=(1+k_{10}X_{11})(1-X_{11})/(k_{11}+1-X_{11})-k_{12}X_{13}X_{11}X_{12}/(k_{13}+X_{11})$ | 0.9 |
| $X{}_{12}$ | | cyclin-CDK | $dX_{12}/dt=k_{14}-(k_{15}+k_{16}X_{12}+k_{17}X_{14})X_{12}$ | 0.01 |
| $X{}_{13}$ | | Mass of the cell | $dX_{13}/dt=k_{18}X_{13}(1-X_{13}k_{19})$ | 5 |
| $X{}_{14}$ | | Protein 27 | $dX_{14}/dt=k_{20}-k_{21}(X_{13}/m_{*}+k_{23})X_{14}$ | 0 |
| $X{}_{15}$ | | RBNP | $dX_{15}/dt=k_{23}-(k_{24}+k_{23}X_{12})X_{15}$ | 1 |
