# Supplementary material for: Multi-scale agent-based brain cancer modeling and prediction of TKI treatment response: Incorporating EGFR signaling pathway and angiogenesis
Source: BMC Bioinformatics. 2012 Aug 30;13:218. doi: 10.1186/1471-2105-13-218 (PMC3487967; doi:10.1186/1471-2105-13-218)
Supplement: Additional file 4 — Table A4. Parameter in cell-cycle pathway. [file 1471-2105-13-218-S4.doc]

**Table 4** Parameters in the cell-cycle set of ODEs [10, 21].

| **Symbol** | **Value** | **Unit** | **Symbol** | **Value** | **Unit** |
| --- | --- | --- | --- | --- | --- |
| $k{}_{10}$ | 10 | *Min^-1^* | $k{}_{19}$ | 10 | *DC* |
| $k{}_{11}$ | 0.04 | *DC* | $k{}_{20}$ | 0.007 | *DC* |
| $k{}_{12}$ | 35 | *Min^-1^* | $k{}_{21}$ | 0.01 | *DC* |
| $k{}_{13}$ | 0.04 | *DC* | $k{}_{22}$ | 0.0017-0.0025 | *DC* |
| $k{}_{14}$ | 0.04 | *DC* | $k{}_{23}$ | 0.01 | *DC* |
| $k{}_{15}$ | 0.4 | *Min^-1^* | $k{}_{24}$ | 0.1 | *DC* |
| $k{}_{16}$ | 1 | *Min^-1^* | $m_{*}$ | 10 | *DC* |
| $k{}_{17}$ | 0.25 | *Min^-1^* | *thr_1_* | 0.004 | *DC* |
| $k{}_{18}$ | 0.01 | *Min^-1^* | *thr_2_* | 0.05 | *DC* |
