# Supplementary material for: Multi-scale agent-based brain cancer modeling and prediction of TKI treatment response: Incorporating EGFR signaling pathway and angiogenesis
Source: BMC Bioinformatics. 2012 Aug 30;13:218. doi: 10.1186/1471-2105-13-218 (PMC3487967; doi:10.1186/1471-2105-13-218)
Supplement: Additional file 5 — Table A5. Parameters of microenvironmental PDEs in the model. [file 1471-2105-13-218-S5.doc]

**Table 5** Parameters of the micro-environmental PDEs in the model. Their values are taken from the literature and have been rescaled to our model.

| **Symbol** | **Value** | **Unit** | **Description** | **Equation** | **Reference** |
| --- | --- | --- | --- | --- | --- |
| $D{}_{G}$ | $6.7\times10^{-7}$ | $cm^{2}s^{-1}$ | Diffusion coefficient of glucose | (4) | [10] |
| $D{}_{C}$ | $8\times10^{-5}$ | $cm^{2}s^{-1}$ | Diffusion coefficient of oxygen | (5) | [10] |
| $D{}_{T}$ | $5.18\times10^{-7}$ | $cm^{2}s^{-1}$ | Diffusion coefficient of TGF$\alpha$ | (6) | [10] |
| $D{}_{V}$ | $2.9\times10^{-7}$ | $cm^{2}s^{-1}$ | Diffusion coefficient of VEGF | (7) | [19] |
| $S_{T}$ | $0.2$ | $nM/h$ | Secretion rate of TGF$\alpha$ | (6) | [11] |
| $S_{V}$ | $0.6$ | $nM/h$ | Secretion rate of VEGF | (7) | Estimated |
| $U{}_{G}$ | $0.28$ | $mmol/h$ | Uptake rate of glucose | (4) | [10] |
| $U{}_{C}$ | $6.25\times10^{-8}$ | $DC/h$ | Uptake rate of oxygen | (5) | [32] |
| $P{}_{G}$ | $3\times10^{-5}$ | $cm/s$ | Permeability of glucose | (4) | Estimated |
| $P{}_{C}$ | $3.0\times10^{-4}$ | $cm/s$ | Permeability of oxygen | (5) | [32] |
| $P{}_{V}$ | $0.1\times10^{-4}$ | $cm/s$ | Permeability of VEGF | (7) | [19] |
| $G_{m}$ | $57$ | $nM$ | Maximum concentration of glucose | A(1) | [10] |
| $G_{a}$ | $17$ | $nM$ | Normal concentration of glucose | A(1) | [10] |
| $T_{m}$ | $9010.55$ | $nM$ | Maximum concentration of TGF$\alpha$ | A(3) | [11] |
| $C_{m}$ | $0.0025$ | $DC$ | Maximum concentration of oxygen | A(2) | [10] |
| $C_{a}$ | $0.0017$ | $DC$ | Minimun concentration of oxygen | A(2) | [10] |
| $Tki_{m}$ | $10$ | $\mu M$ | Maximum concentration of TKIs | A(6) | Assumed |
| $Tki_{a}$ | $0.5$ | $\mu M$ | Minimum concentration of TKIs | A(6) | [27] |
| $\beta$ | $0.05$ | $DC$ | Production rate of fibronectin | (8) | [19] |
| $\gamma$ | $0.1$ | $DC$ | Uptake rate of fibronectin | (8) | [19] |
| α | 2600 | *Cm^2^s^-1^M^-1^* | Chemotatic coefficient | (9) | [19] |
| λ | 986 | *Cm^2^s^-1^M^-1^* | Haptotatic coefficient | (9) | [19] |
| $r$ | 10 | $\mu m$ | Average radii of micro blood vessel | (4) | [33] |
| $k_{V}$ | $1.6667\times10^{-10}$ | $DC$ | Constant of VEGF chemotactic sensitivity | (9) | [19] |
