# Supplementary material for: Multi-scale agent-based brain cancer modeling and prediction of TKI treatment response: Incorporating EGFR signaling pathway and angiogenesis
Source: BMC Bioinformatics. 2012 Aug 30;13:218. doi: 10.1186/1471-2105-13-218 (PMC3487967; doi:10.1186/1471-2105-13-218)
Supplement: Additional file 6 — Text A1. Equations describing the Initial distribution of glucose, oxygen, TGFα, VEGF and fibronectin. [file 1471-2105-13-218-S6.doc]

**Additional Materials**

**Multi-scale Agent-Based Brain Cancer Modeling and**

**Quantitative Prediction of TKI Treatment Response:**

**Incorporating EGFR Signaling Pathway and Angiogenesis**

Xiaoqiang Sun, Le Zhang, Hua Tan, Jiguang Bao, Costas Strouthos and Xiaobo Zhou

The initial distributions of glucose, oxygen and TGFα are described by the equations (1-3) [10], with
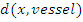
 being the distance from location $x=(x_{1},x_{2})$to line
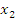
=0 where the parent vessel is located.
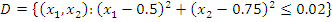
 is the domain where initial tumor cells are distributed.
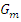
 and
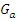
 are the maximum and minimum concentrations of glucose in the blood, respectively;
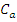
 and
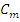
 are the minimum and maximum oxygen concentrations, respectively.
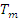
 and
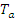
are the maximum and minimum TGFα concentrations, respectively.
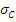
 and
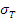
 are the parameters controlling the dispersion of glucose, oxygen and TGFα, respectively (Table 5).

Equation (4) describes the initial concentration of VEGF arising from a small circular tumor [19], where $r=((x_{1}-0.5)^{2}+(x_{2}-0.75)^{2})^{1/2}$ is the distance from location *x* to the center of the initial tumor, and $v$ is a positive constant. The initial condition of fibronectin equation is given by equation (5) [19].

We assume that TKIs preexists throughout the host tissue domain. The initial distribution of TKIs is described by equation (6) below.

$G_{0}=G_{a}+(G_{m}-G_{a})\exp(-2d^{2}(x,vessel)/\sigma_{G}^{2})$, (1)

$C_{0}=C_{a}+(C_{m}-C_{a})\exp(-2d^{2}(x,vessel)/\sigma_{c}^{2})$, (2)

$T_{0}=T_{m}\exp(-2d^{2}(x,vessel)/\sigma_{T}^{2})$. (3)

$V_{0}=\left\{\begin{matrix} 1, \\ (v-r)^{2}/v-0.1, \end{matrix}\right.$ $\begin{matrix} 0\leq r\leq0.1, \\ 0.1\leq r\leq1. \end{matrix}$ (4)

$F_{0}=0.75\exp(-x_{1}^{2}/0.45)$, (5)

$TKI_{0}=Tki_{m}+(Tki_{m}-Tki_{a})\exp(-2d^{2}(x,vessel)/\sigma_{Tki}^{2})$. (6)
