# Supplementary material for: Multi-scale agent-based brain cancer modeling and prediction of TKI treatment response: Incorporating EGFR signaling pathway and angiogenesis
Source: BMC Bioinformatics. 2012 Aug 30;13:218. doi: 10.1186/1471-2105-13-218 (PMC3487967; doi:10.1186/1471-2105-13-218)
Supplement: Additional file 7 — Figure A1. Tumor induced angiogenesis and vascular tumor growth without TKI treatment. [file 1471-2105-13-218-S7.doc]

**Additional Figure 1.** Tumor induced angiogenesis and vascular tumor growth at different time intervals. The tumor develops tree branching microvasculature stimulated by VEGF. Different colors denote different tumor cell states: dead (black), quiescent (cyan), proliferative (pink), active (blue), and endothelial (red).
