# Supplementary material for: Multi-scale agent-based brain cancer modeling and prediction of TKI treatment response: Incorporating EGFR signaling pathway and angiogenesis
Source: BMC Bioinformatics. 2012 Aug 30;13:218. doi: 10.1186/1471-2105-13-218 (PMC3487967; doi:10.1186/1471-2105-13-218)
Supplement: Additional file 8 — Figure A2. Vascular tumor growths with concentration change of fibronectin. [file 1471-2105-13-218-S8.doc]

**Additional Figure 2.** Vascular tumor growth in the presence of fibronectin at different time intervals. The concentration of fibronectin (dark blue in the background) is high near the parent vessel and low away from it. The yellow color represents the sprout tip endothelial cells. Different cell phenotypes are depicted by different colors: active (yellow), quiesent ( cytan), proliferative (green), apoptotic (blue).
