# Supplementary material for: Multi-scale agent-based brain cancer modeling and prediction of TKI treatment response: Incorporating EGFR signaling pathway and angiogenesis
Source: BMC Bioinformatics. 2012 Aug 30;13:218. doi: 10.1186/1471-2105-13-218 (PMC3487967; doi:10.1186/1471-2105-13-218)
Supplement: Additional file 10 — Figure A4. Various tumor cell numbers without TKI treatment. [file 1471-2105-13-218-S10.doc]

**Additional Figure 4.** Different tumor cell numbers at different time intervals. Depicted from left to right and from top to bottom are the numbers of active cells, dead cells, migratory cells, proliferative cells, vessel cells and average change rate of PLCγ.
