# Supplementary material for: Multi-scale agent-based brain cancer modeling and prediction of TKI treatment response: Incorporating EGFR signaling pathway and angiogenesis
Source: BMC Bioinformatics. 2012 Aug 30;13:218. doi: 10.1186/1471-2105-13-218 (PMC3487967; doi:10.1186/1471-2105-13-218)
Supplement: Additional file 12 — Figure A6. Vascular tumor growth in the presence of fibronectin with TKIs treatment. [file 1471-2105-13-218-S12.doc]

**Additional Figure 6.** Vascular tumor growth in the presence of fibronectin and with TKI treatment at different time intervals. The different colors represent cells types as already defined in the Additional Figure2.
