# Supplementary material for: Multi-scale agent-based brain cancer modeling and prediction of TKI treatment response: Incorporating EGFR signaling pathway and angiogenesis
Source: BMC Bioinformatics. 2012 Aug 30;13:218. doi: 10.1186/1471-2105-13-218 (PMC3487967; doi:10.1186/1471-2105-13-218)
Supplement: Additional file 13 — Figure A7. The concentration change of glucose, oxygen, TGFα and VEGF with TKI treatment. [file 1471-2105-13-218-S13.doc]

**Additional Figure 7.** The concentrations of glucose, oxygen, TGFα and VEGF with TKI treatment at different time intervals.
