# Supplementary material for: Multi-scale agent-based brain cancer modeling and prediction of TKI treatment response: Incorporating EGFR signaling pathway and angiogenesis
Source: BMC Bioinformatics. 2012 Aug 30;13:218. doi: 10.1186/1471-2105-13-218 (PMC3487967; doi:10.1186/1471-2105-13-218)
Supplement: Additional file 14 — Figure A8. The concentration change of TKIs shown at 60, 150, 240 and 300 hours. [file 1471-2105-13-218-S14.doc]

**Additional Figure 8.** TKIs concentration at different time intervals. The TKIs delivered the vascular network, then penetrates the vessels and diffuses towards the tumor.
