# Supplementary material for: Multi-scale agent-based brain cancer modeling and prediction of TKI treatment response: Incorporating EGFR signaling pathway and angiogenesis
Source: BMC Bioinformatics. 2012 Aug 30;13:218. doi: 10.1186/1471-2105-13-218 (PMC3487967; doi:10.1186/1471-2105-13-218)
Supplement: Additional file 15 — Figure A9. Various tumor cell numbers with TKI treatment. [file 1471-2105-13-218-S15.doc]

**Additional Figure 9.** Different tumor cell numbers versus time from simulations with TKI treatment. Depicted from left to right, from top to bottom are the numbers of active cells, dead cells, migratory cells, proliferative cells and quiescent cells.
